# Development of a Web-Based Health Care Intervention for Patients With Heart Disease: Lessons Learned From a Participatory Design Study

**DOI:** 10.2196/resprot.7084

**Published:** 2017-05-17

**Authors:** Birgitte Noergaard, Marianne Sandvei, Nina Rottmann, Helle Johannessen, Uffe Wiil, Thomas Schmidt, Susanne S Pedersen

**Affiliations:** ^1^Research Unit of User PerspectivesDepartment of Public HealthUniversity of Southern DenmarkOdenseDenmark; ^2^Department of PsychologyUniversity of Southern DenmarkOdenseDenmark; ^3^Research Unit of General PracticeDepartment of Public HealthUniversity of Southern DenmarkOdenseDenmark; ^4^Center for Health Informatics and TechnologyMaersk Mc-Kinney Moller InstituteUniveristy of Southern DenmarkOdenseDenmark; ^5^Department of CardiologyOdense University HospitalOdenseDenmark; ^6^Erasmus Medical CenterRotterdamNetherlands

**Keywords:** participatory design, heart disease, telemedicine, workshops, end user involvement, mobile health, mHealth

## Abstract

**Background:**

The use of telemedicine technologies in health care has increased substantially, together with a growing interest in participatory design methods when developing telemedicine approaches.

**Objective:**

We present lessons learned from a case study involving patients with heart disease and health care professionals in the development of a personalized Web-based health care intervention.

**Methods:**

We used a participatory design approach inspired by the method for feasibility studies in software development. We collected qualitative data using multiple methods in 3 workshops and analyzed the data using thematic analysis. Participants were 7 patients with diagnosis of heart disease, 2 nurses, 1 physician, 2 systems architects, 3 moderators, and 3 observers.

**Results:**

We present findings in 2 parts. (1) Outcomes of the participatory design process: users gave valuable feedback on ease of use of the platforms’ tracking tools, platform design, terminology, and insights into patients’ monitoring needs, information and communication technologies skills, and preferences for self-management tools. (2) Experiences from the participatory design process: patients and health care professionals contributed different perspectives, with the patients using an experience-based approach and the health care professionals using a more attitude-based approach.

**Conclusions:**

The essential lessons learned concern planning and organization of workshops, including the finding that patients engaged actively and willingly in a participatory design process, whereas it was more challenging to include and engage health care professionals.

## Introduction

Telemedicine is the use of information and communication technologies (ICT) to deliver health care at a distance [1]. During the last decades, use of telemedicine technologies in the health care sector has increased substantially, with the use of such technologies continuing to evolve. Such telemedicine approaches have the potential to improve patients’ outcomes, offer remote access to health care, and reduce health care costs [1]. The benefits of telemedicine are widely described, including better communication between health care professionals and patients [2], improved quality of life for patients with heart failure [3], reduced hospitalization frequency and length [4], and increased patient empowerment [5,6]. However, it is important to cautiously assess the full impact of introducing telemedicine interventions [7], including the long-term effects and whether such interventions may have adverse effects in subsets of patients [8].

In parallel with this development in the health care sector, there is a growing interest in participatory design methods when developing telemedicine approaches to health care [9]. Participatory design entails the involvement of end users in the design and implementation of technology, with participatory design methods having been used for developing ICT-based services for different purposes and patient populations [10-12]. Ideally, participatory design should be initiated as early in the design phase as possible and in a setup that involves representatives of all major end user groups [13]. User involvement will increase the likelihood of creating technological solutions that meet the needs and preferences of end users in their specific social and organizational contexts [11,12]. In turn, this is likely to enhance efficacy and to ensure that the product makes a difference to patients and the health care system, while also increasing the likelihood of successful implementation in clinical practice [14].

### The ACQUIRE Project

Our study was carried out as part of the ACQUIRE (Advance the Quality of Life and Care of Patients with Heart Disease) project, aiming to contribute to the development and design of a Web-based health care intervention for patients with heart disease with an implantable cardioverter-defibrillator.

The overall objective of the ACQUIRE project is to evaluate the clinical efficacy and cost effectiveness of the health care intervention as an add-on to usual care as compared with usual care alone using a multicenter randomized controlled trial design. The health care intervention is based on a modular platform provided by an international information technology company. The platform is made available to end users as a Web app that supports both browsers on computers and mobile platforms, such as tablets. During the participatory design process, the health care intervention was extended with tools to support this specific study.

The health care intervention is expected to increase patient empowerment and to enable patients with heart disease and an implantable cardioverter-defibrillator to live a better life with their device and their disease. The intervention will encourage patients to become co-managers of their own disease and enable patients to routinely track their health status and symptoms of anxiety and depression, flag deteriorations early on, initiate interactions with the implantable cardioverter-defibrillator outpatient clinic, and give patients access to appropriate self-management advice and tools via a Web-based platform. The platform also serves as a tool to inform health care professionals in a timely manner about changes to patients’ symptoms to allow early intervention, support shared decision making, and provide more tailored treatment.

### Aim

The aim of this paper is to present lessons learned from a Danish case study involving both patients with heart disease and health care professionals with respect to the development of a personalized Web-based health care intervention (the ACQUIRE project).

## Methods

### Design

The design basis for our participatory design study was the above-mentioned generic telemedicine platform that offered a variety of health tracking and self-management tools, of which we focused on patients’ monitoring of health status (questionnaire), symptoms of anxiety and depression (questionnaire), and communication support. Furthermore, we included self-management tools in terms of online health information, debate forums, and diaries. We tested the platform on both tablet and computer interfaces.

The methodological approach was inspired by the method for feasibility studies in software development (MUST) [13,15]. MUST is a meta-method for participatory design, especially used for information technology projects. We used MUST as a conceptual framework, as the method describes 4 guiding strategies for action for the participatory process: (1) well-defined concepts to help understand and frame the intervention, (2) principles ensuring user involvement, first-hand experience with the technology, and anchoring, (3) techniques for data collection, and (4) organization of the participatory design process [13,15]. In our process, we used these strategies as guiding principles in the planning of workshops and application of workshop results.

### Workshops and Recruitment

To ensure first-hand experience with the platform (MUST guiding strategy 2), we organized a total of 3 workshops from December 2015 to January 2016. The duration of each workshop was 2 hours, with the following generic structure (MUST guiding strategy 4): (1) presenting the aim and procedure of the workshop, as well as participants’ different roles during the workshop; (2) introducing a specific topic on the platform (eg, health status tracking and self-management tools); (3) testing the specific topic through hands-on exercises; (4) discussing the pros and cons of the specific topic, both in groups and in plenary; and (5) anchoring end users’ first-hand experiences by documenting key learnings.

**Table 1:** Patients participating in the participatory design workshops.

Sex	Age (years)	Duration of disease (years)	Experience with tablets and computers	Workshop 1 (tablet)	Workshop 2 (computer)	Workshop 3
Male	74	Several	Some experience with computer	Yes	No	Yes
Female	71	17	Some experience with computer	Yes	No	Yes
Female	46	16	Experienced user of tablet and computer	Yes	No	Yes
Female	66	1	Experienced user of computer	No	Yes	Yes
Male	76	6	Experienced user of computer	No	Yes	Yes
Female	57	1	Experienced user of computer	No	Yes	Yes
Male	66	11	Experienced user of computer	No	Yes	Yes

We aimed to include a diverse group of patients in terms of age, sex, and the duration that patients had lived with their heart disease. It was also essential that the participating patients had both communicative skills and sufficient motivation and capabilities to engage actively in the process together with health care professionals and systems architects. We included 7 patients with a diagnosis of heart disease: 3 men and 4 women. The patients’ mean age was 65 years, and they had different levels of user experience with tablets and computers. Table 1 shows further details describing these patients.

We also intended to include physicians and nurses from the 5 participating hospitals to ensure ownership among the health care professionals who, in the end, would be working with the health care intervention. However, during the process, we had to give up on the idea of involving health care professionals in all workshops, as they were not able to take part due to time constraints. Thus, 2 nurses and 1 physician participated in the first workshop, all of whom were women and representing 2 hospitals.

In addition to patients and health care professionals, 2 systems architects, 3 moderators (researchers experienced in qualitative methods and participatory design), and 3 observers (a PhD student, a master’s student, and a project nurse) were present during all workshops. The main responsibility of the moderators was to facilitate hands-on exercises and discussions, while the responsibility of observers was to make observational notes during hands-on exercises. The systems architects’ main role was to present the concept and assist with technical issues (MUST guiding strategy 1). One moderator facilitated the overall procedure of the workshops. Textbox 1 presents the major content and attending participants in the 3 workshops.

**Overview of content and attending participants in the workshops.**

**Workshop 1: Tracking tools and related functions, tablet interface**

- Introduction to workshop and presentation of tracking tools (questionnaires assessing health status and symptoms of anxiety and depression)
- Hands-on exercises (in groups of 1 patient, 1 health care professional, 1 systems architect, 1 moderator, and 1 observer)
- Group discussions (groups of patients only and of health care professionals only)
- Plenary discussion

Participants: 3 patients, 2 nurses, 1 physician, 2 systems architects, 3 moderators, and 3 observers

**Workshop 2: Tracking tools and related functions, computer interface**

- Introduction to workshop and presentation of tracking tools (questionnaires assessing health status and symptoms of anxiety and depression)
- Hands-on exercises (in groups of 1 patient, 1 systems architect, and either 1 moderator or 1 observer, or both)
- Plenary discussion

Participants: 4 patients, 2 systems architects, 3 moderators, and 3 observers

**Workshop 3: Self-management tools**

- Introduction to workshop and presentation of self-management tools, including health information material on heart disease (text and video), a debate forum on Facebook for patients with heart disease, and an online letterbox for medical advice and support from health care professionals
- Hands-on exercises (in groups of 2 patients, 1 systems architect, and either 1 moderator or 1 observer, or both)
- Plenary discussion

Participants: 7 patients, 2 systems architects, 3 moderators, and 3 observers

### Data Collection and Analysis

Prior to each workshop, we gave a detailed script to moderators, observers, and systems architects describing the various steps of the workshop and responsibilities of all stakeholders. The script also included a short semistructured interview guide for the hands-on exercises, as well as introductions to group and plenary discussions.

During the hands-on exercises, the observer made observational notes focusing on end users’ main challenges during the exercises and their attitudes to the topic under discussion. Group and plenary discussions were audiorecorded and transcribed verbatim (MUST guiding strategy 3).

Following each workshop, we analyzed the themes of the observational notes and transcriptions of discussions. We coded the transcripts and observational notes according to the themes of the interview guide and then identified core themes, mapping end users’ experiences and attitudes. The main results of each workshop were presented in work-in-progress reports addressed to the principal investigator (SSP) and systems architects who were deciding on the further development of the health care intervention.

### Formal Approvals and Ethical Considerations

The investigation conforms with the principles outlined in the Declaration of Helsinki [16]. A project nurse identified and contacted patients at the outpatient clinic of the Odense University Hospital to inform them orally and in writing about the participatory design process. Health care professionals were identified and contacted by the principal investigator (SSP).

The workshops were approved by the principal investigator, and all participants signed a consent form accepting participation, that discussions would be audiotaped, and that data would be published afterward.

We submitted the study protocol to the Regional Committees on Health Research Ethics for Southern Denmark, who indicated (email communication, October 19, 2015) that ethical committee approval is not required by Danish law on ethics related to health research (§ 14, 1). We also sought and obtained permission to proceed with the study from the Danish Data Protection Agency under the umbrella agreement of the University of Southern Denmark (2015-57-008).

## Results

Findings from the project fall into 2 parts. First, we focus on the outcomes of the participatory design process. This includes the actual contributions from end users—patients and health care professionals—to the development and customization of the generic telemedicine platform, as well as related work practices. Second, we describe the experiences gained from the process and reflect on the added value of conducting the participatory design research process from the researcher’s perspective.

### Outcomes of the Participatory Design Process: Users’ Input Into Customizing the Telemedicine Platform and Service

Based on the thematic analysis of users’ input into the customization and refinement of the platform and related services, we categorized the findings into 6 major areas: (1) users’ feedback on the ease of use of tracking tools and presentation of monitoring results, (2) users’ feedback on platform design, (3) users’ feedback on terminology, (4) insights into users’ monitoring needs, (5) insights into patients’ ICT skills and preferences, and (6) insights into patients’ preferences for self-management tools.

#### Users’ Feedback on Ease of Use of Tracking Tools and Presentation of Monitoring Results

Although participants generally found that the tracking tools (questionnaires assessing health status and symptoms of anxiety and depression) were easy to use, they had several suggestions for refinement of the system’s procedural flow. For instance, participants found it inappropriate that they could not correct their previous responses in the questionnaire and agreed that it should be possible to modify responses while completing the questionnaire. Similarly, they pointed out the inappropriateness of only being allowed to fill in their comments in questionnaire textboxes *before* giving a response, and accordingly agreed that it would be more suitable if the system allowed comment boxes to be filled in both *before* and *after* responding to a particular question. There was a general agreement on the relevance of including a visual indicator that would state how far they had come with completion of questionnaires (eg, question 5 out of 20).

#### Users’ Feedback on Platform Design

In terms of the presentation of questionnaire results, participants agreed on the necessity of including visual indicators supporting patients in the interpretation of graphs depicting their health status, symptoms of anxiety and depression, and accumulation of scores over time. One of the suggestions from the health care professionals was to add intuitive indicators, such as smiley faces on the y-axis, to make the interpretation of a rising or falling curve indisputable. Other design issues commented on by patients and health care professionals related to the number of topics presented on the welcome page (eg, display of completed questionnaires and future questionnaires to be filled in), which participants suggested should be reduced to create a better overview. Some patients argued that the font size used was too small considering the target group of elderly patients. Participants agreed that a built-in function in the system to enlarge font sizes would make the platform more accessible to (elderly) visually impaired patients.

#### Users’ Feedback on Terminology

Participants agreed that it was a core issue to use terminology uniformly throughout the platform. They stressed the importance of applying terms commonly used in the information material from the outpatient clinic rather than more technical terms, foreign words, or abbreviations (eg, participants preferred the use of “heart disease” to “congestive heart failure”).

#### Insights Into Users’ Monitoring Needs

Patients expressed significant differences in their attitudes toward tracking their health status and symptoms of anxiety and depression. While none of the patients found it problematic to answer the health status questionnaire, some patients indicated that the anxiety and depression questionnaire was either irrelevant or that it “got too close” and thus was too confrontational. Moreover, patients’ attitudes toward their monitoring needs differed substantially. Discussions during the workshops suggested that it was primarily patients with a new diagnosis, or patients whose health status had recently deteriorated, who were in favor of routine monitoring, as this contributed to an increased sense of security. Patients who had lived with the disease for many years had a more critical attitude toward the health status tracking tools and argued that there was a risk of inducing unnecessary concerns and focusing too much on disease.

#### Insights Into Patients’ Information and Communication Technologies Skills and Preferences

The lessons learned from the first and second workshops also related to patients’ ICT skills. In workshop 1, patients tested the platform on tablets, while in workshop 2, they completed tests on computers. The fact that 2 out of 3 patients who participated in the first workshop hardly had any experience with tablets revealed that many of the taken-for-granted functionalities of the tablet interface were not evident to these elderly patients (eg, scrolling menus related to response options in questionnaires). One of the very basic lessons learned from this workshop was that, for the implementation of Web-based interventions to succeed, it is paramount that the system use a responsive design to support technology that is familiar to individual patients.

#### Insights Into Patients’ Preferences for Self-Management Tools

The focus of the third workshop was to generate ideas for self-management advice and tools to be added to the platform. In this workshop, patients tested the different types of tools presented in Textbox 1 on various platforms. Workshop discussions revealed patients’ different attitudes to these self-management tools. Most of the patients favored having heart disease-related information easily accessible directly on the platform. Some of the patients already had experience with online debate forums and liked the idea of adding this tool to the platform, while, to other patients, online patient-to-patient contact did not seem like a relevant option. None of the patients had experience with receiving medical advice or support through an online letterbox and argued that they would prefer to contact their contact person at the outpatient clinic if they needed professional advice. Thus, a key finding from our workshops is that, given the heterogeneity of patients with heart disease and their different needs and preferences, it is important that not only the interface but also the content can be targeted to the individual patient.

### Value of Participatory Design in the Development of Telemedicine Services

As described in the methods section, our a priori strategy was to bring together patients, health care professionals, and systems architects in the same workshops to create mutual learning and understanding. We achieved this goal only in workshop 1, as no health care professionals participated in workshops 2 and 3. We found the combination of patients and health care professionals indeed appropriate and widely unproblematic. Prior to the workshop, we were concerned whether patients would be heard and therefore we structured group discussions for patients and health care professionals separately. However, all participants seemed aware of their responsibilities for everyone to be heard, both during group discussions and during plenary discussions. We found that the most distinct difference between patients and health care professionals was in their different ways of approaching the participatory process: patients actively engaged in the process and contributed with experience-based input, whereas health care professionals were more likely to observe and help patients and thus contribute with input of a more authoritarian and health care professional character. Furthermore, health care professionals’ contributions referred to a large extent to how they figured the patients could handle the telemedicine platform and less to how they could integrate the platform in their own work practices in the outpatient clinic.

As mentioned above, in workshops 2 and 3, we only had patients and systems architects present. Because in workshop 1 we found the interaction between patients and health care professionals to be less dynamic than we had expected, the process regarding customization of the platform with only patients and systems architects present was just as fruitful.

Recruitment of health care professionals appeared to be a key challenge during the process of conducting workshops. Despite persistent efforts to adapt the planning of workshops to the health care professionals’ work schedule (eg, conducting condensed workshops of no more than 2 hours, in different geographic areas, or at the end of a workday), we only partially succeeded in getting them involved; it remains unclear to us whether this was due to workload, lack of interest, or the way the participatory design process was introduced to them. We saw that the health care professionals who actually participated were engaged and showed genuine interest during the workshop. We might have more successfully involved health care professionals by conducting individual interviews; this would, on the other hand, have compromised mutual learning from the dynamics of the workshops.

We also learned that it is crucial to have systems architects present during the workshops, both because they contribute technical knowledge and because it is highly instructive for them to see their product being tested in practice, primarily in relation to the elderly patients and their challenges using the tablet interface.

## Discussion

Our study aimed at understanding end users’ priorities and critical processes in a participatory design process involving patients with heart failure and health care professionals in the customization of a Web-based health care intervention.

The definition of participatory design is broad and slightly ambiguous, although participatory design should *ideally* be initiated in the early phases of the design process [17], as mentioned in the introduction. In our study, we dealt with an existing platform, health status tracking, and self-management tools and, thus, our process is more likely to be a collaborative evaluation [17] or participatory customization process [2]. On the other hand, the MUST approach as a conceptual framework for participatory design comprises tailoring off-the-shelf, ready-made products for specific target groups [13]. Nevertheless, participatory design involves “more than having a voice;” it involves affecting the outcome by “having a say” [13]. When it comes to how our workshops actually helped shape the platform, it is more a question of “how” than of “what”—more of how to customize the usability of the specific platform than of deciding what it takes to empower and encourage patients. This customizing process around “how” is, according to this terminology, “having a say.”

During the process, we faced several challenges; identification of the patient target group and challenges related to engaging health care professionals became core challenges. Regarding patients, representing the population’s diversity was a challenge even though we aimed to include patients representing the population’s heterogeneity in terms of age, sex, and the duration of their disease. Although this approach was methodologically appropriate, we only partly succeeded. Traditionally, some degree of homogeneity in the target group for participatory design is recommended [17]. On the other hand, the heterogeneity of our workshop participants clearly showed that both the intervention and the platform are not “one size fits all;” patients have different needs and preferences. Furthermore, the participatory design process contributed to identifying the target group that would benefit the most from the intervention, namely patients with a relatively recent diagnosis with some experience with tablets or computers.

We had expected health care professionals to engage fully in all 3 workshops, as the participating hospitals had agreed to test the platform. Nevertheless, engaging health care professionals appeared to be a challenge, and we had to adjust our expectations and the aim of including health care professionals in all workshops, and thus compromising the possibilities for mutual learning during the participatory design process [13]. But, because the few participating professionals seemed reluctant to test the platform themselves and contributed more attitude-based input, as opposed to patients’ experience-based inputs, we did not see quite the level of interaction and dynamics between patients and professionals that we had expected. Some argue that a hospital setting is an obvious place for conducting participatory design, as both patients and health care professionals are ready at hand [13]. This is not a lesson learned from our study; despite the geographical proximity of health care professionals, they might have multiple reasons for not participating.

A point of concern regarding telemedicine in general is the risk of introducing unwanted outcomes [8], and our workshops revealed that this could also be a risk of the ACQUIRE health care intervention. In particular, patients with many years of experience with heart disease mentioned the risk of the routine health status tracking inducing unnecessary concerns and focusing too much on disease; they expressed that they were less preoccupied with their heart disease and that they had moved on. This point of view was also reflected in patients’ preferences regarding terminology. Other researchers found that patients prefer “heart problems” to “heart disease” [18], whereas our participants preferred “heart disease” to “congestive heart failure;” in both examples, patients preferred terms signaling fewer problems.

During the workshops, we observed that some patients were reluctant to respond to some of the questionnaire items, as these were of a more sensitive character and they “got too close.” Despite guidance from the moderator, who emphasized that answering these questions was merely considered a simulation, some patients found it difficult to decouple the simulation from their actual situation. Triggering uncertainty regarding trust, sensitive information, and confidentiality may unintentionally influence the workshop outcomes. Hence, in line with the recommendations from Petrova et al [19], our workshops show the importance of ensuring that participants are aware that they are participating in the study to advance the development of a care intervention, and not to provide first-hand patient-specific data.

### Conclusion and Lessons Learned

Our customizing rather than participatory design process provided valuable knowledge of end users’ views on a Web-based health care intervention. In addition, we learned some essential lessons concerning the planning and organization of workshops.

We found that end users involved in developing telemedicine interventions should reflect the specific target group that will finally use the intervention.

We also found that broad inclusion taking into account the heterogeneity of the patient group contributes multiple perspectives and nuances, especially regarding ease of use and interaction with health care professionals. Furthermore, through the process of conducting workshops, it became clearer for whom the intervention was most relevant. Including both patients and health care professionals revealed different perspectives, with patients using an experience-based approach and health care professionals using a more attitude-based approach. We also learned that patients engaged actively and willingly in the process, whereas it was more challenging than we had expected to include and engage health care professionals.

### Implications for Practice and Research

- Broad inclusion contributes multiple perspectives.
- Through the process of conducting workshops, it becomes clearer for whom an intervention is most relevant.
- Patients contribute with an experience-based approach.
- Professionals contribute with an attitude-based approach.
- Patients engage willingly in the participatory design process.
- It is challenging to engage health care professionals.

In conclusion, we recommend 2 important steps for this line of research. First, it is relevant to explore health care professionals’ values and preferences in relation to engaging in participatory design processes to improve their engagement. Second, future research should work with a larger and more representative sample to generate more generalizable knowledge in order to inform best practices.

## Abbreviations

ACQUIRE: Advance the Quality of Life and Care of Patients with Heart Disease
ICT: information and communication technologies
MUST: method for feasibility studies in software development
